# A Long-Term Engagement with a Social Robot for Autism Therapy

**DOI:** 10.3389/frobt.2021.669972

**Published:** 2021-06-16

**Authors:** Nazerke Rakhymbayeva, Aida Amirova, Anara Sandygulova

**Affiliations:** ^1^ Department of Robotics and Mechatronics, School of Engineering and Digital Sciences, Nazarbayev University, Nur-Sultan, Kazakhstan; ^2^ Graduate School of Education, Nazarbayev University, Nur-Sultan, Kazakhstan

**Keywords:** robot-assisted therapy, human-robot interaction, social robots, autism spectrum disorder, attention deficit hyperactivity disorder

## Abstract

Social robots are increasingly being used as a mediator between a therapist and a child in autism therapy studies. In this context, most behavioural interventions are typically short-term in nature. This paper describes a long-term study that was conducted with 11 children diagnosed with either Autism Spectrum Disorder (ASD) or ASD in co-occurrence with Attention Deficit Hyperactivity Disorder (ADHD). It uses a quantitative analysis based on behavioural measures, including engagement, valence, and eye gaze duration. Each child interacted with a robot on several occasions in which each therapy session was customized to a child’s reaction to robot behaviours. This paper presents a set of robot behaviours that were implemented with the goal to offer a variety of activities to be suitable for diverse forms of autism. Therefore, each child experienced an individualized robot-assisted therapy that was tailored according to the therapist’s knowledge and judgement. The statistical analyses showed that the proposed therapy managed to sustain children’s engagement. In addition, sessions containing familiar activities kept children more engaged compared to those sessions containing unfamiliar activities. The results of the interviews with parents and therapists are discussed in terms of therapy recommendations. The paper concludes with some reflections on the current study as well as suggestions for future studies.

## 1 Introduction

Long-term or longitudinal studies are essential and desirable research approach in the human-robot interaction field to document changes over time, which can provide more reliable and rich data with relatively high accuracy. Remarkably, the definitions of long-term research are rare. A more relevant interpretation for the current study was proposed by Ployhart and Vandenberg (2010), defining longitudinal research as the study of change and repeated observations. Despite its salient features, long-term research in the context of human-robot interaction is not common, for instance, only 5 out of 96 empirical studies consisted of more than a single session between 2013 and 2015 (Baxter et al., 2016).

Weitlauf et al. (2014), in their meta-analysis, revealed that traditional interaction-based autism treatments usually range from 6 to 16 weeks, while behavioral interventions can last 3–12 weeks. It was, however, stated that hours of intervention does not correlate with its outcomes. However, it is apparent that the earlier the intervention, the better the intervention outcome. Past research on human-robot interaction shows that long-term studies should take 2 months at minimum before participants get accustomed to a new situation or environment (Sung et al., 2009). Yet, we believe that identifying the set terms for longitudinal studies cannot be accurate. Practically, it is demanding to conduct regular experiments with social robots for autism therapy due to technical and specific challenges associated with working with vulnerable population (Daley et al., 2013). In view of this, we agree that the long-term interaction is defined based on the number of sessions and the length of time allocated to each session (Leite et al., 2013). Particularly, Leite et al. (2013) suggested that the long-term interaction occurs in the time point when the novelty effect diminishes. For instance, young children might feel overwhelmed by a robot, which can be overcome over repeated exposure (Belpaeme, 2020). When humans interact with others for a long period, they usually develop a sense of closeness through performing natural behaviours and self-disclosure. In committing to the long-term engagement goals, such an approach can reduce the anxiety caused by the “new normal” people face with a robot and a co-present researcher or therapist. It also entails “in the field” studies which mainly deal with interactions occurring in natural settings (Oertel et al., 2020). For the most part, designing a long-term interaction is a complex undertaking in terms of time and resource for researchers in the field of HRI. Despite the complexity from an engineering standpoint, longitudinal studies are beneficial to thoroughly explore the dynamics of human-robot interaction and track what worked well and what should be done differently. It can occur in various places where a robot can be co-located at home, in hospital, in kindergarten, at school, and so on. It is therefore important that robots can maintain learning function and be durable for long-term use (Shibata, 2004). Provided that such interaction entails a sense of continuity, a long-term relationship may positively affect social bonding between a human and a companionable robot (Baxter et al., 2011).

Our research is driven by the need to create an individualized experience in the robot-assisted therapy for children with diverse forms of autism, committing to bring positive changes in behaviours through long-term engagement. This study aims at examining the effectiveness of robot-assisted autism interventions incorporating various robotic applications which were appropriated to the needs and preferences of children with ASD. As a result of the intervention, we analyzed valence and engagement scores and investigated whether familiar sessions improved children’s valence and engagement from session to session. We suggest that children would enjoy the activities that were previously played with the robot. This paper presents quantitative analyses of a multiple-session study conducted with 11 children with ASD and ADHD. To the best of our knowledge, there is an overall lack of such studies and data that are necessary to overcome shortcomings of the field pointed out by David et al. (2019), Diehl et al. (2012) in their reviews. That would help to draw firm conclusions on the use of social robots in the long run.

## 2 Related Work

The research shows great variability in the number of sessions during autism therapy. A relatively small amount of studies were conducted in a long term perspective. More studies have been conducted in the home and educational environment as well as in hospital facilities and laboratories.

### 2.1 Home-Based Autism Therapy

Home environment is known to be a common example of long-term studies (Baraka et al., 2020). In addition, home-based studies oftentimes deploy autonomous robots that have simplified mechanisms and can function independently in many instances. Considerable amount of in-home intervention is also available in autism research. Pakkar et al. (2019) designed an autonomous and socially assistive robotic (SAR) system for eight children with ASD who performed different educational and socially diverse activities with a robot during 1 month. Children managed to sustain interest in the proposed system and considered the robot as a friend, most probably due to its animal-like appearance and informal language use. The system was perceived positively by both children and their parents as they rated the robot to be friendly and applicable, despite the limitations on the family environment and occasional technical issues. In the study by Clabaugh et al. (2019), hierarchical human-robot learning (hHRL) was introduced to personalize learning experiences of children with ASD aged 3–8 years old. The hHRL framework was regarded to be effective after 100 sessions in the home environment when the majority of participating children showed improvements in target skills and had learning gains in maths. In a quite similar way, Scassellati et al. (2018) conducted a month-long study with 12 children with ASD who played with the Jibo robot six interactive games in the home environment. The study aimed at improving social and emotional understandings, perspective-taking, ordering, and sequencing skills, which became possible with the robot’s capabilities to adapt the game complexity on the basis of the child’s past performance. The daily session lasted for 30 min and involved a triadic interaction with a child, the robot and a caregiver. Children remained engaged throughout the whole period and improved the observed behaviours with caregivers.

### 2.2 Play-Based Autism Therapy

As distinct from this context, long-term studies have been also undertaken, predominantly within the context of educational facilities. As part of the AuRoRa project, a longitudinal study by Robins et al. (2005) investigated the extent to which repeated exposure benefits social behaviours in four children with ASD between 5 and 10 years of age. Throughout several months, Robota was programmed to play a decisive role in the four behaviours—imitation, eye gaze, touch, and child-robot proximity. In the first phase, children were introduced to the robot in the box, which performed dance moves to attract their attention. In later trials, children and robot interacted with the help of the investigator who encouraged children to play imitations games and controlled the robot by WoZ. As a result, children were able to interact with and respond to the robot in an unconstrained environment (Robins et al., 2007). further encouraged the use of play-based environment for autism therapy ranging from solitary to social and cooperative play with other people, including peers, teachers, parents, or carers in schools.

Later François et al. (2009), presented a robot-mediated non-directive play therapy involving six children with autism and a dog-like Aibo robot in the UK-based school. The experiments were conducted once a week, with a maximum of ten sessions in total. Using video recordings of the session, the children’s progress was analyzed based on three dimensions—Play, Reasoning, and Affect. Results have shown that children progressed in a different way and improved at least one out of the three dimensions since each child’s specific needs and abilities were taken into consideration during therapeutic interventions.

In Netherlands, a study by Huskens et al. (2014) used a robot-mediated intervention based on LEGO® therapy to examine the impact of collaborative play behaviour. The population comprised three Dutch sibling pairs, including a typically developing child and a child with ASD. Sibling pairs were randomly assigned to different number of three, four, or five sessions. The dependent variables included collaborative behaviours identified as 1) interaction initiations, 2) responses to questions or instructions from TD siblings, and 3) “play together” actions to achieve a common goal. They had five 30-min sessions once a week, a robot would instruct one child of the sibling pair to be the guide, the other the LEGO® builder. The authors pointed to the non-significant effectiveness of the collaborative play therapy.

### 2.3 ABA-Based Autism Therapy

Inspired by the traditional ABA therapy, Srinivasan et al. (2015) carried out rhythm and robotic therapy intervention with NAO and Rovio. They assessed repetitive and maladaptive behaviours (RMBs) in 36 children with ASD aged 5–12 during early, mid and last intervention sessions. Overall, each interaction lasted for 45 min with a total of 32 sessions in 8 weeks, attended by the three groups—rhythm, robot, or comparison. The authors measured frequencies in the standard time of sensory, positive, and negative affect, and stereotyped behaviours across sessions. Negative affect referred to off-task behaviours such as looking away, distressing mood, while positive affect was measured by time spent on smiling. It was found that the rhythm group reduced negative behaviours after training compared to other groups. Children in the rhythm and robotic groups showed greater affect across all sessions. The authors emphasized that movement/music-based activities sustain joint attention and verbalization in children, thereby creating an enjoyable and socially engaging scenario in rhythm therapy.

Conducted in the Dutch context, Otterdijk et al. (2020) evaluated the long-term child-robot interaction through observing engagement and attention during pivotal response treatment (PRT) with the robot for 20 sessions which were attended by each child. Each game-based PRT session lasted 15–20 min. As it was a part of large scale studies Korte et al. (2020), they hypothesized that children’s engagement and attention decreases throughout the intervention, considering previous research that states that children lose interest and get disengaged over time. Results indicate that children’s engagement with and attention towards the robot and the game did not change over time. Interestingly, children linearly increased their attention towards and engagement with their parent in the therapy over time, while there is no increase in relation to the therapist.

### 2.4 Emerging Issues

One unifying goal for these studies was to create a less intimidating social environment, where children with autism can increase social engagement and communication skills. Socio-emotional bonds with individuals with autism may develop differently than other people’s; therefore, engagement is the key indicator to assess the quality of interaction. In research, the concept of engagement is interpreted differently. Engagement is defined in various contexts and usually includes attention, involvement, interest, immersion, rapport, empathy, and stance used in inextricable and interchangeable fashion (Salam et al., 2016). Notably, a triadic interaction appears to be a desirable setting for robot-child communication since for researchers it was important to transfer social skills to the co-present others. Children with higher social functioning and milder ASD symptoms tend to initiate more spontaneous interaction with the robot (Schadenberg et al., 2017; Kumazaki et al., 2018), and in some cases, they were attracted to the robots as opposed to humans or other agents (Bharatharaj et al., 2017; Cao et al., 2019). The robot personality is another contributing factor to the engagement, for instance, children exhibited significantly greater compliance with the personality-matching robot (Andrist et al., 2015). More essentially, the introduction of socially assistive robots may improve motivation and compliance in rehabilitation settings due to their physical embodiment and ability to use human-like communication channels.

Most studies have raised the issues around the child-robot interaction in autism therapy from a technical perspective, proposing to develop fully/semi-autonomous and sophisticated robotic system (Rudovic et al., 2017; Anzalone et al., 2019; Melo et al., 2019). Robot autonomy is vital to the progress being made in HRI, but we also need to remember its facilitating role in autism therapy. Therapeutic and educational objectives are by far the most defining issues rather than the technology itself (Wood et al., 2019). Moreover, most researchers face methodological constraints, including but not limited to recruitment and retention, a small collection of activities, and experimental barriers. Evaluating user experiences with a social robot in the field scenario requires more original data collection tools than in any other environment (Leite et al., 2013). This also may not be technically feasible as it is demanding to reach generalizable conclusion on the effect of the robotic intervention on children with autism. In addition, interactions based on a similar set of behaviours may seem repetitive and monotonous over an extended time, resulting in decreased user engagement when the novelty effect disappears. It is suggested that personalization can help sustain long-term user engagement when adapted to the user’s characteristics, preferences, and needs. Thus far, a handful of research studies (François et al., 2009; Scassellati et al., 2018; Sandygulova et al., 2019) attempted to design an adaptive social scenario that focuses on many-sided interactive and engaging activities. This gap in research has become our core purpose with the belief that long-term commitment and individualized activities improve the social engagement of children with ASD. Repeated exposure to robot-assisted therapy necessitates research on the dynamics of human-robot interaction. Our work attempts to direct research attention towards individualized approach to fulfil the needs and preferences of children in a socially active and flexible environment with a robot.

## 3 Methodology

This research was approved by the ethics committees of Nazarbayev University and Republican Children’s Rehabilitation Center, Kazakhstan. This study followed the methodological design, as was described in the previous work (Sandygulova et al., 2019). The experiments were carried out in the Republican Children’s Rehabilitation Center where children and their parents are admitted to the center for a 21-days period to undergo therapy that includes traditional methods of autism therapy (art, music therapy, and others). On the first day of their stay, children underwent diagnostic assessment provided by the doctors while therapists learned about each child’s individual differences.

### 3.1 Robot Activities

Due to a large variety of behavioural differences in ASD and ADHD children, a large number of robot applications were implemented and fine-tuned from session to session, as shown in our previous work (Sandygulova et al., 2019). These activities targeted joint attention, imitation, turn-taking skills as well as emotional well-being of the participants. Video demonstrations of the activities can be found at the link: bit.ly/rat-nu.

#### 3.1.1 Dances

The main goal of this block of activities was to support children’s emotional well-being. Children were encouraged to repeat dance movements after the robot or simply watch and listen to the robot. We used off-the-shelf dances, namely, “Ganghnam style” and “Macarena.” They varied in terms of tempo and pace to diversify the range of the movements and music. Dances were programmed to be launched using scripts in a Wizard-of-Oz approach and lasted for about 1–2 min.

#### 3.1.2 Songs

Similar to the Dances, this block was programmed to support children’s emotional well-being. We created a simple choreography for the robot to perform movements corresponding to the rhythm of the songs provided to us by the music therapist. They were with different tempo and pace: “Clock,” “Painter,” “Helper,” “Spider,” “Fixers,” “Mothers,” “Wash your hands,” “Heroes,” “Beautiful,” “Red Apricot,” “Maria,” and “Tanya.” Some of these songs were only used with either Russian or Kazakh speaking children, while the others were used for both groups. Song activities were launched upon touching the robot’s tactile sensors on its head and both arms and feet. Each song took approximately 1–2 min.

#### 3.1.3 Emotions

The first activity of this block targets joint attention for children to direct their attention to the pictures placed on the left side and on the right side of the robot. Children were encouraged by the therapist to express emotions and repeat after the robot. The robot demonstrated five emotions in sequential order (happy, sad, surprised, bored, and interested). Each display of emotion was accompanied by audio (e.g. laughter sound, crying sound, etc). There were two printed images for each emotion: an image of a situation and an image of a child experiencing that emotional state. Thus, for each emotion, the robot pointed at the photo with a situation, performed animation, and told what emotion it felt, and then pointed at the photo with a child showing that emotion. As an example, the robot said that it feels happy to receive gifts by pointing at the photo with a gift and performing a happy exclamation with a sound and a hand raising gesture. Then, it points at the photo with a smiley child while saying that this child is also feeling happy to receive gifts. Each emotion was introduced on separate days and had a total duration of 3 min.

Another activity of this group aims to practice social actions, imitation, and turn-taking skills. We programmed a set of simple and social non-verbal communicative actions such as clapping, high-five, peace sign, handshake, sending a kiss, hugging, yawning, and others. After each action demonstration, the robot asked children to repeat the action with their parents and/or with a therapist and waited for 10 s to repeat it. All of these social actions were accompanied by a situation-based introduction of the action (e.g. “I am yawning when I feel sleepy,” “I am clapping when I feel happy”) in order to engage children. In total this activity lasted for 4 min.

#### 3.1.4 Touch Me

This activity aims to develop skills of tactile contact when interacting with the robot. It also intends to teach vocabulary related to body parts. The NAO robot requests to touch one of the body part. Despite the use of the verb “touch,” we also used other verbs in the same request, e.g. “pat on my head,” “brush my head,” “tap my blue toes on my right foot,” “stroke a blue spot on my right hand,” and so on. When a child touches the correct tactile sensor, the robot expresses its praise, and applauds. Because of children with different forms of ASD, we built this activity to be without logic and negative response. When a child touched the wrong body part, the robot kept quiet before the correct response. Additionally, the difference was only between pressing its head, arms and feet. Right and left body parts were calculated as the correct answers. This activity did not have an exact duration and was terminated *via* the script.

#### 3.1.5 Storytelling

To improve the concentration and imagination skills of children, we programmed three storytelling activities, in which the robot acted out popular fairy tales such as “The Bun,” “The Turnip,” and “The Cockerel.” The storytelling was supported in two languages using animated movements and sounds when necessary. Each story lasted around 1.30–2 min.

#### 3.1.6 Imitation

To target imitation skills, we programmed the robot to perform verbal and nonverbal behaviours based on different themes. They were “Transports,” “Animals,” and “Sports.” “Transports” activity included four animations of transports (car, motorcycle, airplane, and boat). “Animals” activity comprised four animated behaviours of animals such as gorilla, mouse, elephant, and horse. Similarly, “Sports” activity consisted of well-known sports such as basketball, football, archery, and hockey. The robot had to imitate transports and animal through gestures, movements, and sounds. It then encouraged children to imitate in the same way. Additionally, each animation was accompanied by a printed image to maintain joint attention. Each animation was repeated randomly. Therefore, this block of activities did not have an exact duration and was stopped *via* the script.

### 3.2 Hypothesis and Conditions

The following hypotheses were formulated in order to test to what extent the individualized sessions in a multi-session study will lead to increased engagement and valence scores.

• H1: Children will increase their engagement and valence scores when interacting with the robot over multiple sessions.
• H2: Using activities familiar to each child will lead to increased overall engagement from session to session.

### 3.3 Recruitment

As all families arrived on the same day at the Rehabilitation Center, we waited 2 days for parents and children to get acquainted with the system at the hospital. On the third day of their stay with the help of therapists and nurses, we arranged a meeting with all families in the common area to demonstrate the robot.

During this demonstration, the robot was muted and turned on in live mode. The research purpose, data collection procedures, risks, and benefits were explained by one of the researchers. We distributed the informed consent forms to the parents, and they were given time to ask questions. All researchers were open to respond to any question put by parents. Parents were allowed to take the printed consent forms to carefully read at their preferred pace, and we also provided our contact details in case something was unclear. Our meeting lasted approximately 2 h in the same venue. The majority of parents signed the consent forms within 2 h, while some parents returned the signed forms the following day. The parents or caregivers provided their written informed consent to participate in the interviews. Also, with the written consent, each session was video recorded using a standard web camera for later annotation and evaluation.

At the end of the meeting, we asked for parents’ contact numbers upon permission to include them in the group chat for further correspondence regarding the session timetable. Noticeably, all parents agreed to join the group chat since they have known each other before the study.

### 3.4 Participants

The study involved 11 children (one girl) aged 4–11 years old diagnosed with ASD on the premises of the Republican Children’s Rehabilitation Center. In particular, all children were diagnosed with ASD, while seven out of 11 children were diagnosed with both ASD and ADHD. At the time of the study, the mean age of the children was 6.1 years (SD = 2.7 years). Table 1 presents the participants’ characteristics provided to us by the therapists and parents.

**TABLE 1:** Children’s demographics and other characteristics (ADOS-2 score, ASD form, presence of co-occurring ADHD, verbal or nonverbal autism), compliance to therapists’ instructions, and the number of attended sessions.

Id	Age group	Sex	ADOS-2	ASD form	ADHD	Verbal	Compliance	Sessions
C1	5–6	M	6	Moderate	−	−	✓	10
C2	10–11	M	9	Severe	✓	✓	✓	9
C3	7–8	M	9	Severe	✓	−	−	9
C4	5–6	M	8	Severe	✓	−	−	8
C5	5–6	M	8	Severe	✓	−	−	8
C6	5–6	M	6	Moderate	✓	−	−	8
C7	5–6	M	7	Moderate	✓	−	−	8
C8	10–11	M	9	Severe	−	✓	✓	7
C9	6–7	M	8	Severe	−	−	✓	7
C10	5–6	F	5	Moderate	✓	−	✓	7
C11	4–5	M	5	Moderate	−	✓	−	7

A therapist employed by the Rehabilitation Center performed ADOS-2 scoring test with every child at the beginning of their stay at the center (Lord et al., 2012). The therapist has around 7 years of experience working at the center and performs the test regularly. ADOS-2 is a test that evaluates the characteristics of a child’s communication, social interaction, and play skills. The purpose of the ADOS test is to observe the child’s behavior during games, tasks, and conversation in sequential order. The severity of autism-related symptoms is assessed by comparative points: 3–4 corresponds to a mild range, 5–7 to a moderate range, and 8–10 scores to a severe range. There were five children with the moderate form of autism and six children with the severe form. Eight children were nonverbal and did not speak, except for a few words. Compliance in Table 1 means that children typically followed the instructions issued by the therapist.

### 3.5 Setup

**FIGURE 1:**
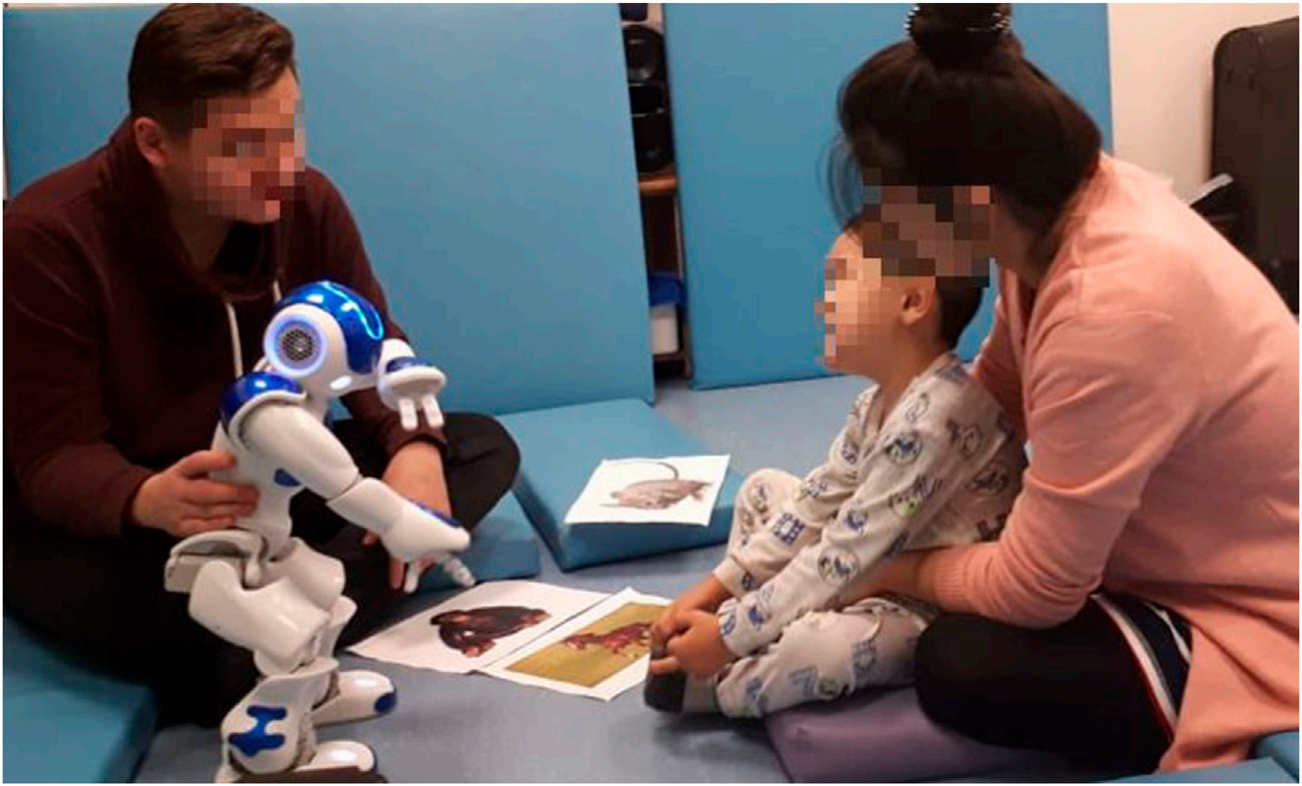
Experimental setup.

All sessions took place in a small-sized and furniture-free room, having only sports mats on the floor and walls. The robot and a child were positioned on the floor which allows them to keep eye contact and move freely. Figure 1 displays the setup. Two recording cameras were placed in the room: the first was near the child on the mat, while the second was hung on the wall to record the whole room. The robot was connected *via* a wi-fi router. The researcher, with a computer, sat behind the mats, and controlled the session by launching applications.

### 3.6 Procedure

The children attended a series of 15-min sessions with the NAO robot. Every child participated in at least seven sessions (each session was on separate days). It was planned for a maximum of 10 robot-assisted sessions, but due to personal reasons (e.g. a child falling asleep or having swimming activity) some children missed their sessions, thus there is a variation in the number of sessions between the participants (Table 1 presents the number of sessions attended by each child). Each session lasted for around 15 min, but it was planned in advance to stop the session if a child loses interest or wants to leave the room. Parents were invited to attend the sessions but it was not compulsory for them to be present and it was up to each parent to decide how to act. Some parents provided prompts and repeated target behaviours with children, while others simply observed.

When sessions ended, we conducted semi-structured interviews with parents or caregivers and asked them to compare their children’s first reaction to the robot and what they could compare it to. We moved on to ask if they observed any changes in the child’s behaviour after each session and after all sessions. We also documented some personal information about each child: autistic traits and preferences for toys, play activities, and technology usage skills. Eventually, we concluded our interviews by asking for further suggestions and reflective feedback on the research. All questions can be found in the **Appendix**. However, due to the lengths concerns, we only provide some of the insights regarding the therapy recommendations received from parents and therapists.

### 3.7 Sessions: Process of Familiarity and Liking

At the beginning of each session, the robot greeted children and introduced itself: “Hello, my name is NAO. We are going to play with you, dance, sign songs, and listen to fairy tales. What would you like to start with?” Since all robot activities were unfamiliar to children, the therapist asked them and their parents (in case when child is non-verbal) what they would like to start with. Based on their responses and general information about each child (e.g. sensitivity to sounds), the therapist made her choice for the first session. Overall, all robot activities were performed with all children throughout all sessions. However, there were some children who refused to play certain activities. For example, C10 rejected the activities like “Emotions” and “Imitations.” C11 also refused to play “Emotions” similar to C10. Additionally, C1, C2 and C7 objected to “Dances” activity as they were sensitive to sounds.

Robot activities were introduced gradually depending on each child’s reaction and performance. The role of a human therapist was to choose an activity for the robot based on a child’s reaction to it and inform the researcher what activity to launch via a command-line terminal. Since it was unfeasible to follow the same interaction flow with every child on account of diverse conditions of ASD and ADHD, the order and type of applications were customized on the basis of observations and therapist’s feedback. Therefore, for the third session onward, the order and type of activities were customized according to each child’s performance and observed preference. For example, some children did not like dance and song activities, while others liked only dances and songs but disliked touching the robot. Table 2 presents robot activities for each child in each session. For each child, as can be seen from the table, there was a gradual shift towards the most favourite activities in the last few sessions. For example, C11 particularly liked “Touch Me” and “Storytelling” but disliked “Songs” and “Dances.” Similarly, C2 did not like song and dance activities but enjoyed “Storytelling,” “Emotions,” and “Imitations.” C2 did not prefer “Touch Me” compared to C11. “Storytelling” was the least liked activity for C1, C5, C9. Children C7, C8, and C9 preferred to play activities like “Emotions” and “Imitations.” C1 and C10 had a shared dislike for “Touch Me” and enjoyed “Songs” and “Dances”. Overall, more popular activities for most children were “Songs” and “Dances” (C3, C4, C5, C6, C7, C8, C9, and C10) with a slight disinterest in “Storytelling” and preference for “Emotions” between children.

**TABLE 2:** Robot activities for each child in each session.

Child	Session	Robot activities and frequencies
C1	S1	Songs (x23)
	S2	Songs (x3), Touch Me (x1)
	S3	Touch Me (x1), Songs (x10), Imitations (x1)
	S4	Songs (x12), Imitations (x1)
	S5	Imitations (x2), Songs (x4), Storytelling (x1)
	S6	Songs (x7), Emotions (x1)
	S7	Emotions (x1), Imitations (x1), Songs (x7)
	S8	Imitations (x2), Emotions (x1), Songs (x7)
	S9	Emotions (x2), Storytelling (x1), Imitations (x1), Songs (x4)
	S10	Emotions (x2), Imitations (x1), Songs (x9)
C2	S1	Songs (x2)
	S2	Storytelling (x1), Touch Me (x1), Imitations (x1)
	S3	Storytelling (x3), Imitations (x2)
	S4	Storytelling (x2), Imitations (x2), Songs (x1), Emotions (x1)
	S5	Storytelling (x2), Imitations (x2), Emotions (x1), Songs (x1)
	S6	Storytelling (x3), Imitations (x2), Emotions (x1)
	S7	Storytelling (x2), Imitations (x2), Touch Me (x1), Songs (x1), Emotions (x1)
	S8	Storytelling (x2), Emotions (x1)
	S9	Storytelling (x1), Emotions (x2), Imitations (x2)
C3	S1	Storytelling (x2), Songs (x7)
	S2	Storytelling (x2), Touch Me (x1), Songs (x4)
	S3	Touch Me (x1), Dances (x1), Songs (x3), Imitations (x1)
	S4	Touch Me (x1), Storytelling (x1), Dances (x3), Imitations (x1)
	S5	Storytelling (x1), Songs (x3), Dances (x3), Imitations (x1)
	S6	Storytelling (x1), Dances (x3), Touch Me (x1), Songs (x4)
	S7	Touch Me (x1), Imitations (x1), Songs (x2), Storytelling (x1), Dances (x2)
	S8	Emotions (x1), Songs (x4), Dances (x1)
	S9	Emotions (x1), Songs (x6), Dances (x1)
C4	S1	Songs (x1), Touch Me (x2), Imitations (x1), Dances (x1)
	S2	Touch Me (x1), Imitations (x1), Dances (x1)
	S3	Touch Me (x1), Songs (x2), Storytelling (x1)
	S4	Imitations (x1), Touch Me (x1), Songs (x2), Storytelling (x1)
	S5	Touch Me (x1), Emotions (x1), Songs (x2), Storytelling (x1)
	S6	Imitations (x2), Songs (x3)
	S7	Storytelling (x1), Songs (x4), Imitations (x1)
	S8	Emotions (x1), Songs (x8), Storytelling (x1)
C5	S1	Imitations (x1), Songs (x1)
	S2	Songs (x4), Touch Me (x1), Dances (x2), Imitations (x1)
	S3	Imitations (x1), Storytelling (x1), Songs (x1), Touch Me (x1)
	S4	Imitations (x1), Touch Me (x1), Songs (x6)
	S5	Touch Me (x1), Imitations (x1), Songs (x5)
	S6	Touch Me (x1), Songs (x4), Imitations (x1), Storytelling (x1)
	S7	Emotions (x1), Songs (x4), Touch Me (x1)
	S8	Songs (x10)
C6	S1	Songs (x8), Storytelling (x1)
	S2	Emotions (x1), Imitations (x1), Dances (x1), Touch Me (x1)
	S3	Touch Me (x1), Songs (x2), Storytelling (x1), Imitations (x1)
	S4	Touch Me (x1), Storytelling (x1), Songs (x2), Dances (x1)
	S5	Songs (x10), Imitations (x1)
	S6	Songs (x5), Touch Me (x1)
	S7	Songs (x4), Storytelling (x1), Imitations (x1), Touch Me (x1)
	S8	Songs (x8)
C7	S1	Storytelling (x2), Touch Me (x1), Imitations (x1), Songs (x3)
	S2	Songs (x4), Imitations (x1), Storytelling (x1)
	S3	Storytelling (x1), Imitations (x2)
	S4	Imitations (x2), Songs (x7), Storytelling (x1)
	S5	Imitations (x2)
	S6	Imitations (x1), Songs (x8), Touch Me (x2), Storytelling (x1)
	S7	Imitations (x2), Emotions (x1), Songs (x7)
	S8	Emotions (x1), Songs (x8)
C8	S1	Songs (x8), Storytelling (x1)
	S2	Touch Me (x1), Imitations (x1), Storytelling (x1), Dances (x1)
	S3	Emotions (x1), Dances (x1), Storytelling (x1)
	S4	Storytelling (x2), Touch Me (x1), Dances (x1)
	S5	Imitations (x1), Storytelling (x3), Songs (x3), Dances (x1)
	S6	Touch Me (x1), Storytelling (x2), Emotions (x1), Dances (x1), Songs (x4)
	S7	Songs (x2), Emotions (x1), Dances (x2), Storytelling (x1)
C9	S1	Storytelling (x1), Songs (x3)
	S2	Touch Me(x1), Emotions (x1), Songs (x3), Dances (x1)
	S3	Imitations (x2), Songs (x5)
	S4	Touch Me (x1), Storytelling (x1), Songs (x7), Dances (x1)
	S5	Touch Me(x1), Songs (x11)
	S6	Emotions (x2), Songs (x9)
	S7	Emotions (x1), Songs (x12), Imitations (x1)
C10	S1	Dances (x3)
	S2	Dances (x6), Touch Me (x3)
	S3	Dances (x9), Songs (x5)
	S4	Dances (x5), Touch Me (x3)
	S5	Dances (x2), Songs (x5)
	S6	Songs (x9), Dances (x4), Storytelling (x1)
	S7	Songs (x5), Storytelling (x2)
C11	S1	Dances (x3), Touch Me (x3)
	S2	Touch Me (x1)
	S3	Touch Me (x4), Storytelling (x2), Songs (x1)
	S4	Touch Me (x3), Storytelling (x2)
	S5	Touch Me (x4), Storytelling (x4), Songs (x3), Dances (x2)
	S6	Touch Me (x2), Storytelling (x1)
	S7	Storytelling (x2), Touch Me (x3), Imitations (x4)

A session would be labelled as familiar when it consisted of mostly familiar (previously encountered) activities. When a session had mostly unseen activities, it was labelled as unfamiliar session. This way, all first sessions were labelled as unfamiliar, and then the labelling varied for each child depending on the ratio of familiar vs unfamiliar sessions.

Those children that missed a few sessions had a varying engagement pattern on the following sessions: C6 and C9 had a slightly increased engagement, C5’s engagement score was not affected while C2, C3, C4, C7, and C8 had a slight decrease in engagement. These patterns can be seen in Figure 1.

### 3.8 Video Coding

All videos were recorded with a web camera embedded with a microphone. Two researchers independently coded 50% of the video recordings using the ELAN software. 20% of data was cross-coded by the other researcher. The agreement score on this 20% of data was computed from pair-wise ICC of the coders and equals 82.6%. We followed the same coding strategy as prior works by Kim et al. (2012) and Rudovic et al. (2017): engagement is scored on a 1–5 Likert scale with one corresponding to the child being fully non-compliant (evasive) and five when fully engaged (1-Full Non-compliance, 2-Non-compliance, 3-Several Prompts, 4-One/Two Prompts, and 5-Immediate Reaction), and valence is scored on a 1–5 Likert scale with one corresponding to the child expressing negative emotions and five to positive emotions (1-Cry/Anger/Fear, 2-Sad/Bored, 3-Neutral, 4-Interested, and 5-Happy/Excited).

Kim et al. (2012) coded 10-s long fragments of videos while Rudovic et al. (2017) coded the whole engagement episode to preserve the context (starting with the target task until one of the engagement scores is met). We coded engagement and valence scores relative to the timing of applications. As such, each application had two scores assigned to it: Engagement and Valence.

In addition, we coded eye gaze and engagement duration and then calculated their percentages relative to the overall time of the session (e.g. engagement duration of 3 min out of a 12-min session results in a value of 25%).

### 3.9 Measures

Once we video-coded the interactions, we calculated the following measures:

• $S_{n}Engagement$: mean of engagement scores calculated for each session. There were 10 variables for sessions 1–10.
• $S_{n}Valence$: mean of valence scores for each session. There were 10 variables for sessions 1–10.
• $S_{n}EngagementTime$: the amount of time the child is engaged during one session. This variable is calculated relative to the overall time of the session (e.g. $S_{n}EngagementTime$ is 25% i.e. 3 min out of 12 min-session). There were 10 variables for sessions 1–10.
• $S_{n}EyeGazeTime$: the amount of time the child spent looking at the robot calculated relative to the overall duration of the session. There were 10 variables for sessions 1–10.
• Mean_Engagement: an average of all engagement scores for familiar and unfamiliar sessions (see Section 4.2). There were two variables of this measure type for each category group.
• Mean_Valence: an average of valence scores for familiar and unfamiliar sessions. There were two variables of this measure type for each category group.
• Mean_EngagementTime: the mean of $S_{n}EngagementTime$ that the child was engaged during all sessions. Similarly, there were two variables for familiar and unfamiliar sessions.
• Mean_EyeGazeTime: an average amount of time the child spent looking at the robot during all sessions. Similarly, there were two variables for familiar and unfamiliar sessions.

## 4 Results

A series of one-way repeated measures ANOVA with a Greenhouse-Geisser correction was run on a sample of 11 childrens to determine if there were differences in valence and engagement scores, engagement, and eye gaze durations as a within-subject comparison between different sessions.

### 4.1 Comparison Between Sessions

In order to test H1, we performed comparison of $S_{n}Engagement$, $S_{n}Valence$, $S_{n}EngagementTime$, and $S_{n}EyeGazeTime$ across sessions for all children. The results showed a non-significant difference in the engagement time the children were engaged between S1, S2, S3, S4, S5, S6, S7, S8, S9, and S10 sessions: F(6,60)=3.0,p=0.05. And other three metrics did not demonstrate significant differences (p>0.05) between these sessions. Table 3 demonstrates these results for all children over each session.

**TABLE 3:** Results of mean values for each measurement for all children during each session.

Measurement	S1	S2	S3	S4	S5	S6	S7	S8	S9	S10
Engagement score	2.82 ± 0.63	3.24 ± 0.82	2.86 ± 065	3.12 ± 0.67	3.34 ± 0.67	2.93 ± 0.56	2.82 ± 066	2.93 ± 0.86	3.1 ± 0.83	3.53 ± 041
Valence score	3.21 ± 0.41	3.36 ± 0.43	3.34 ± 0.53	3.45 ± 0.45	3.35 ± 0.42	3.31 ± 0.27	3.38 ± 0.42	3.48 ± 0.54	3.34 ± 0.47	3.58 ± 0.26
Engagement time	56.57 ± 26.3	59.69 ± 24.41	58.02 ± 22.83	69.25 ± 21.58	75.64 ± 20.69	66.4 ± 17.78	59.9 ± 20.88	73.13 ± 24.03	75.88 ± 19.54	78.38 ± 20.14
Eye gaze time	53.83 ± 21.17	70.72 ± 18.53	69.08 ± 18.87	70.3 ± 18.98	69.7 ± 10.49	68.3 ± 18.46	42.56 ± 12.79	71.24 ± 25.56	73.8 ± 19.66	79.4 ± 12.28

### 4.2 Familiar vs Unfamiliar

To test H2, we conducted a repeated measures ANOVA with a Greenhouse-Geisser correction on a sample of 11 children. The sessions were labelled as unfamiliar when they included unseen activities, while those sessions that consisted of activities that children were comfortable with were labelled as familiar sessions.

**FIGURE 2:**
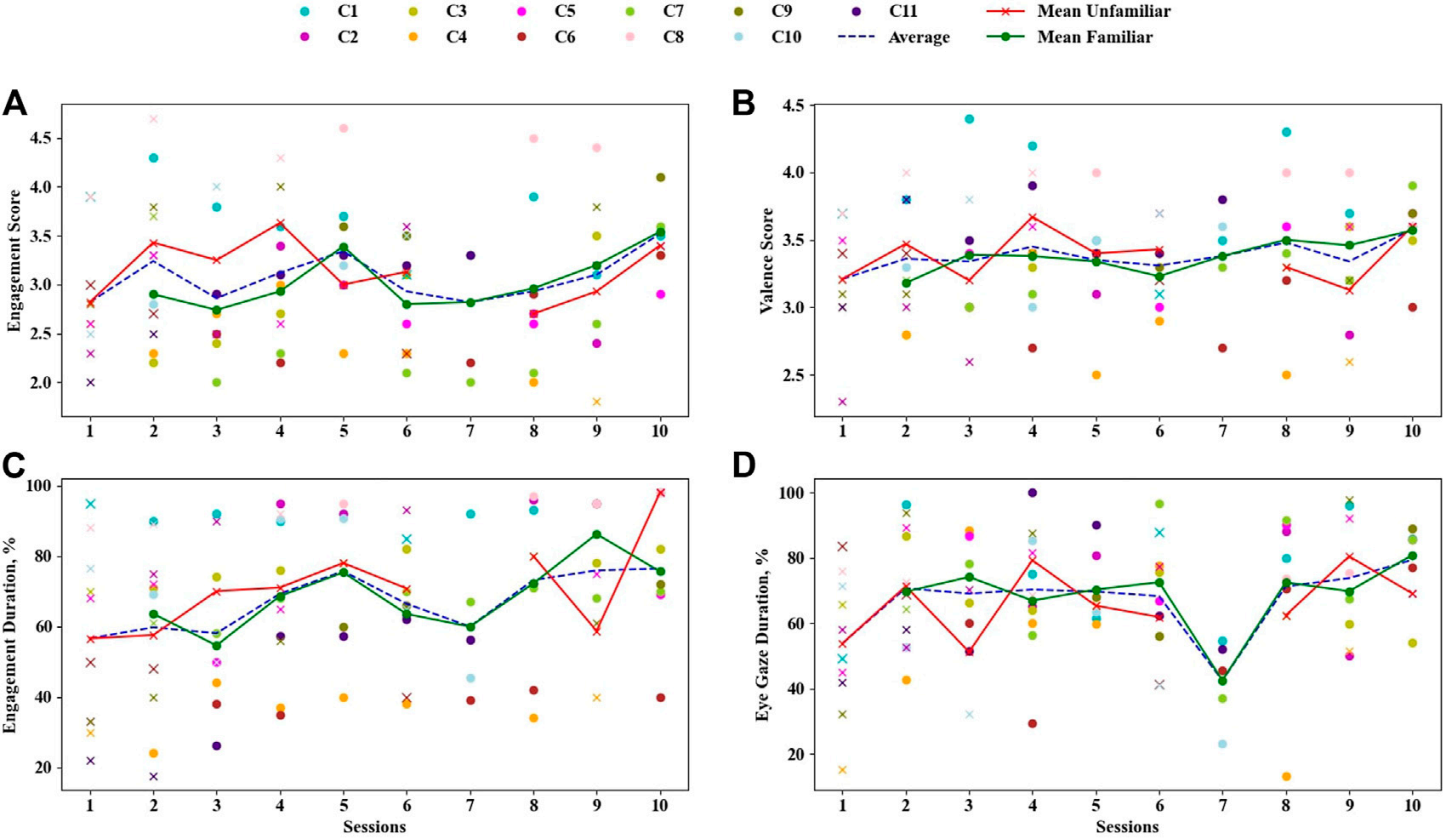
Engagement and valence scores, engagement, and eye gaze durations for each child on each session. Familiar sessions are labelled with circle and unfamiliar sessions are labelled with cross (x) sign. Each colour represents a child.

We found that there were statistically significant differences in engagement duration as follows: the mean engagement score during familiar sessions was significantly higher (3.11±0.24) compared to unfamiliar sessions (2.94±0.22): F(1.507,10.546)=4.678,p=0.043; a mean engagement duration of familiar sessions was significantly higher (70.87±8.51) than unfamiliar sessions (69.98±10.1): F(2.384,16.689)=3.446,p=0.049; a mean eye gaze duration of familiar sessions was significantly higher (75.2±6.82) compared to unfamiliar sessions (65.57±8.84): F(2.087,14.609)=5.232,p=0.018. Figure 2 demonstrates these results in familiar and unfamiliar sessions for each child.

### 4.3 Interviews: Therapy Recommendations

Overall, five parents and two therapists provided their recommendations with regard to activities and robot behaviours during therapy. It should be noted that P1, P4, P5, and P7 could not reflect on potential improvements. The audio recordings of P10 and P11 were lost and could not be retrieved.

Although the parents seemed to be satisfied with the therapy itself, they would like the robot to have more active behaviours. P2 proposed to improve the interactive behaviours of the robot by having a more lively interaction: “There should be more live communication. Only those children who are interested in the robot interacts with it” (P2). Quite similarly, another parent expressed her wish to add name recognition and develop active behaviours in the robot: “The robot should call children by their names like Dima and Vanya. It should be more active, for example, kick a ball and do other kinds of movements” (P3).

Developing cognitively demanding and educational activities was another recommendation provided by P6: “It would be good to have more cognitive activities with visual cards, for example, showing different colours and figures” (P6). Likewise, P9 considered that “the robot should teach counting or distinguish between colours.” In addition, P8 advised using “more poems and songs for verbal children who can repeat after the robot.”

The robot-assisted autism therapy has been a new professional experience for the two therapists. The first therapist shared her opinion about the child-robot interaction scenario: “They should be left alone to observe their behaviours from outside” (T1). The second therapist emphasized that it is equally important to not force children to interact with the robot, referring to one parent: “She (mother) was pushing her (to play with the robot). She should not have done it” (T2). She also mentioned that “children with autism do not like to wait,” which may negatively affect their behaviours during therapy. Both therapists gave examples of future activities such as “tapping xylophone” and “kicking a ball” to support imitation and turn-taking skills. They also recognized the importance of “a long-term triadic interaction consisting of a child with autism, a typically-developing child (e.g. a sibling) and the robot” (T1/T2).

## 5 Discussion

Overall, our study suggests that children with autism remained relatively engaged when interacting with the robot over a prolonged period of time. We found marginally significant difference in engagement results, which support the H1 partially. Although there was no significant difference in engagement duration between the first and the last session, it is notable that the proposed intervention kept the children engaged over multiple sessions. Similarly, valence scores and eye gaze time did not reveal any significant differences between the labelled sessions. Nevertheless, it also means that children did not lose interest in the interaction.

Our second hypothesis is concerned with the relationship between engagement and children’s preference of either familiar or unfamiliar activities. The mean engagement duration and the mean eye gaze duration followed by the mean engagement in familiar sessions were significantly higher as compared to unfamiliar ones. This allows us to accept H2. In line with other researchers (Castellano et al., 2009; Kim et al., 2012), it is worth interpreting children’s liking and positive affect towards some activities as a tangible embodiment of the engagement. In other words, children were more engaged, positive, and focused when we used activities preferred by each child, as observed by the human therapist. According to Korte et al. (2020), children’s experiences stimulated positive feelings when the child-robot interaction was based on meaningful game scenarios. Focusing on communication skills (Srinivasan et al., 2016), emphasized that high engagement levels may be triggered due to activities capitalized on children’s predilections. Thus, it seems mutually beneficial to design an autism intervention depending on each child’s needs and skills, revealing a higher level of individual differences between autistic children in a robot-assisted play (François et al., 2009).

In general, we found interesting results about the long-term engagement during the child-robot interaction in autism therapy. First, we found no significant increase in the engagement rate of children in spite of using multi-purposeful activities. Second, children preferred familiar activities over unfamiliar ones, meaning that they were more engaged with the activities they felt comfortable with. Past research on regular child-robot interaction emphasizes the need for variation and novelty during an interaction to overcome predictability and boredom (Kanda et al., 2007; Coninx et al., 2016). However, this does not work the same way for children with autism. One possible explanation is that individuals with autism tend to prefer routine and sameness and do not like changes (Sevin et al., 2015). In this regard, this core feature of autistic disorder responds to the question of why children on the autism spectrum were attached to familiar activities.

Another way to increase the level of engagement and the benefit of the autism therapy with robots is to advance robotic systems towards autonomy. Past research (Huijnen et al., 2016; Melo et al., 2019; Pakkar et al., 2019) sought to develop adaptive and autonomous robotic systems that reinforce RAT sessions by understanding the child’s individual and social cues and providing broad and personalized experiences. That being said, there is also a concern that such robots cannot respond to the child appropriately in a long-term context due to physical limitations and ethical considerations (Wood et al., 2019). Despite such constraints, we need to continue seeking new scenarios and applications that are capable of bringing positive impact into the area of autism research. The robot’s role as an assistant and a mediator in autism therapy should be further supported by creating adaptive and socially engaging tasks and treatment design.

Lastly, we received valuable feedback from parents and therapists. Their suggestions include endowing NAO with more active and personalized behaviours such as playing an instrument, performing free movement, and calling the children by name. Previous research also highlights the importance of welcome message such as name recognition and hi-five for the quality of interaction with a robot (Tozadore et al., 2016). As of activities, we became aware of the importance of educational content and skills that can be taught by the robot. Besides, it is recommended to manage and increase the amount of time for interaction with a robot similar to traditional therapies. Furthermore, children should be given some time to interact with a robot alone in the room so that others can not intervene. For instance, a few parents insisted on interacting with and repeating after the robot. Taken together, this consideration calls for strategies to optimize the operating time of robots and thus minimize human intervention (Navarro et al., 2011).

## 6 Conclusion

This long-term study applied a quantitative approach with the aim to investigate the social engagement of 11 children aged 4–11 years old diagnosed with ASD and ADHD in robot-mediated interventions. It measured engagement and valence scores and duration using the individualized set of robotic applications, which were specifically designed to initiate social skills. Our findings suggest: 1) it is possible to sustain engagement in children with autism and/or ADHD when interacting with a robot over multiple sessions; 2) children are better engaged and focused during robot-mediated sessions when activities are responsive to each child’s preferences and liking. For the HRI community, the necessity for the use of different activities to increase engagement of children with autism remains open and requires further consideration. It becomes clear in this study that there should be more active and learning-oriented activities in which a child and a robot provide similar inputs to the interaction. While we are a bit far away from presenting more convincing results, our proposed autism intervention makes an encouraging contribution to the long-term HRI.

We intend to analyse teachable moments (times when a child is engaged) and maximize learning events at those times. We also plan to study other factors that could increase engagement and thereby find out ways of taking advantage of those variables to enhance engagement. For further refinement, new methods and active play scenarios will be designed for robot-mediated therapy for children with different autism symptoms.

## Data Availability

The datasets presented in this article are not readily available because participants did not consent to future re-use of their video and interview data by other researchers. Requests to access the datasets should be directed to anara.sandygulova@nu.edu.kz.

## Appendix 1

### 1 Interview Questions

1. What do you think about robot-assisted therapy as an autism therapy for your child?
2. What could you compare your child’s reaction to a robot with? Was the reaction similar to having a toyor, perhaps, seeing an animal?
3. Have you noticed any changes in your child’s behavior after each session?
4. Do you have any recommendations for the improvement of robot behaviors? Any ideas for potential applications?
5. Would your child attend such therapies in the future?
6. Does he/she attend this therapy on his/her own will or do you insist on attending?
7. What’s his/her attitude towards technology? Does he/she use gadgets?
8. What’s his/her favourite activity that keeps engaged?
9. Are there any differences between traditional and robot-assisted therapy? Have you noticed them?
